# Supplementary material for: Chemically Stable Group IV–V Transition Metal Carbide Thin Films in Hydrogen Radical Environments
Source: J Phys Chem C Nanomater Interfaces. 2024 Oct 22;128(43):18524–33. doi: 10.1021/acs.jpcc.4c04822 (PMC11533205; doi:10.1021/acs.jpcc.4c04822)
Supplement: Supplementary file 1 — jp4c04822_si_001.pdf [file jp4c04822_si_001.pdf]

Chemically Stable Group IV-V Transition Metal Carbide Thin Films in Hydrogen Radical Environments

Abdul Rehman[†*], Robbert W.E. van de Kruijs[†], Wesley T.E. van den Beld[†], Jacobus M. Sturm[†], and Marcelo Ackermann[†]

[†]*Industrial Focus Group XUV Optics, MESA+ Institute for Nanotechnology, University of Twente, Drienerlolaan 5, 7522NB Enschede, the Netherlands*

* E-mail: a.rehman@utwente.nl

Supplementary Information

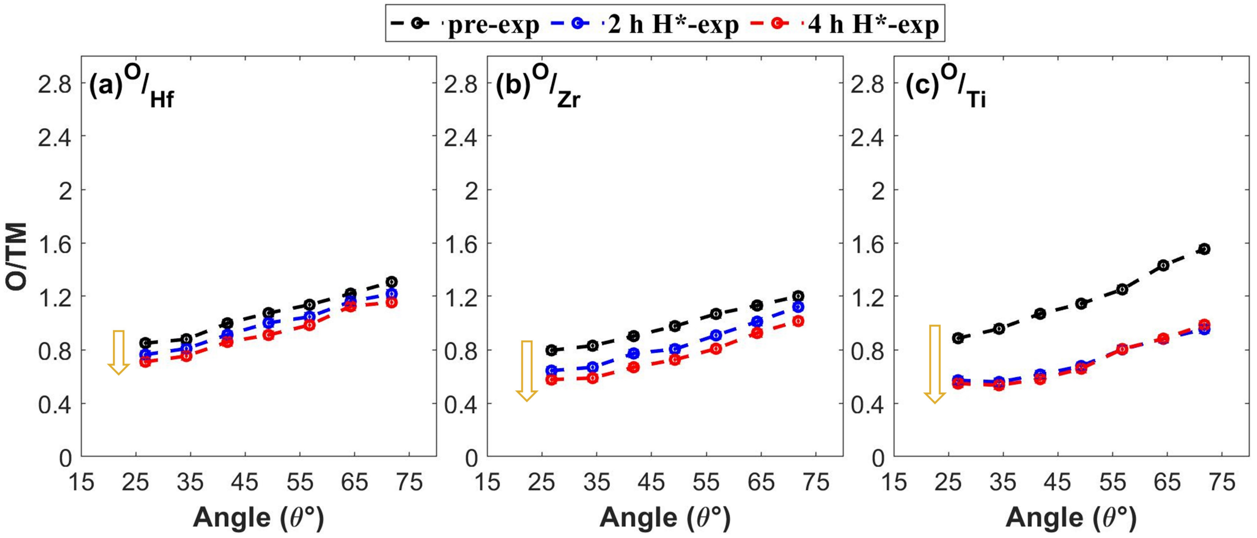

Figure S1. O/TM ratio as a function of θ in the pre-exposed (in black), 2 h H*-exposed (in blue), and 4 h H*-exposed (in red); (a) HfC, (b) ZrC, and (c) TiC samples exposed to H* at 1000 K. A lower O/TM ratio in the TiC sample than in the HfC and ZrC samples following 2 h H*-exposure indicates a stronger surface de-oxiation in the TiC sample than in the HfC and ZrC samples.

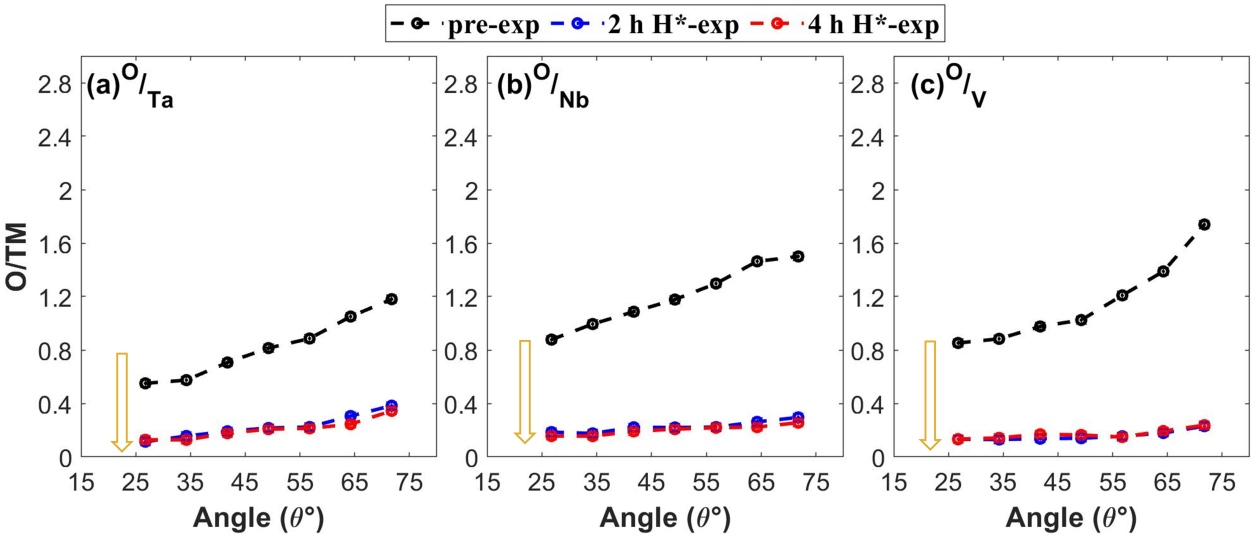

Figure S2. O/TM ratio as a function of θ in the pre-exposed (in black), 2 h H*-exposed (in blue), and 4 h H*-exposed (in red); (a) TaC, (b) NbC, and (c) VC samples exposed to H* at 1000 K. Surface de-oxidation is more pronounced in the TaC, NbC, and VC samples than in the HfC, NbC, and VC samples upon 2 h H*-exposure.

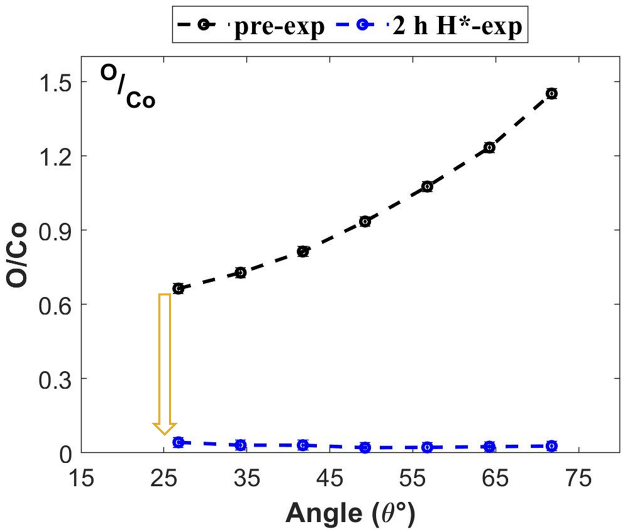

Figure S3. O/Co ratio as a function of θ in the pre-exposed (in black) and 2 h H*-exposed (in blue) Co_2C sample exposed to H* at 500 K. The drop in the O/Co ratio to almost zero suggests a strong surface de-oxidation in the Co_2C sample.

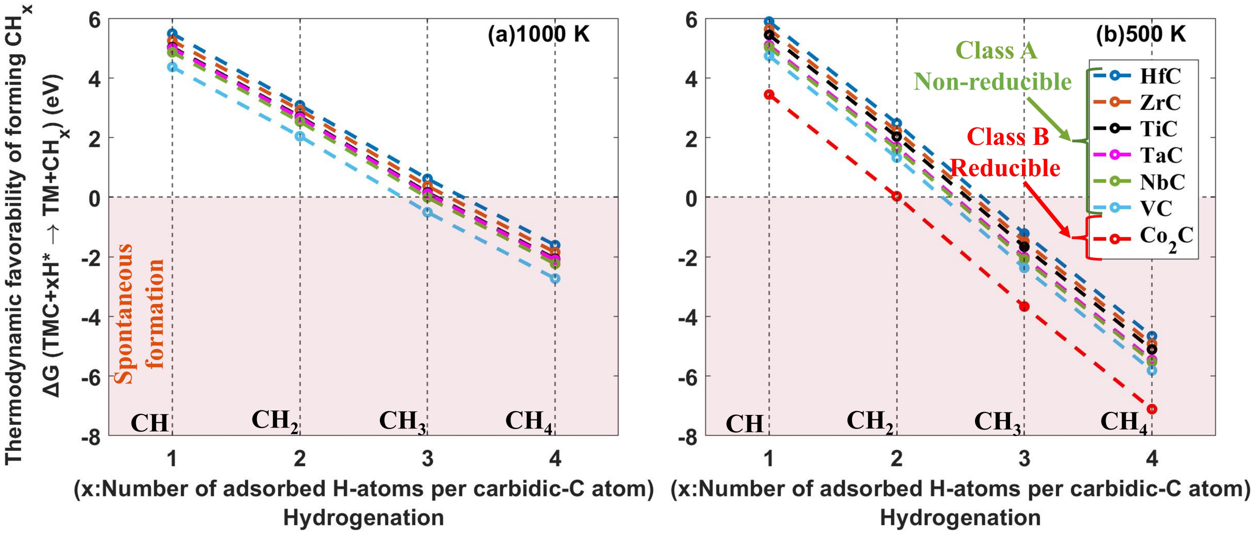

Figure S4. ΔG for the reduction of TMCs per molecule of CH_x ($_x = 1, 2, 3, 4$) calculated at 0.02 mbar for temperatures of (a) 1000 K and (b) 500 K. For group IV-V TMCs, only the formation of CH_4 and CH_3 is thermodynamically feasible under the performed experimental conditions. While, on Co_2C, the formation of CH_2, in addition to CH_3 and CH_4, is feasible.

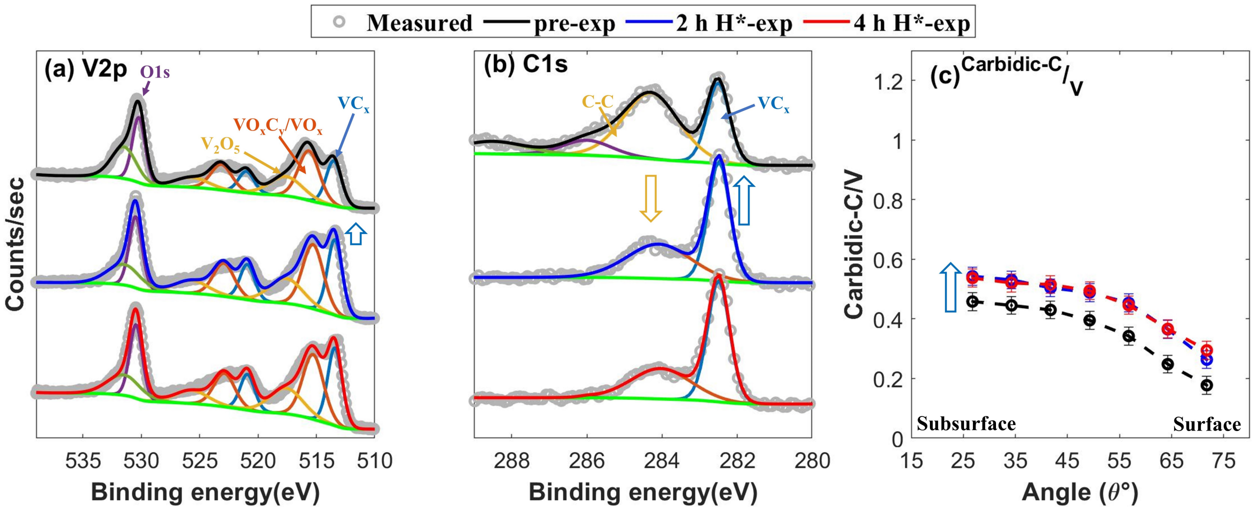

Figure S5. XPS core level TM and C1s spectra taken at $\theta = 34.25°$, along with carbidic-C/TM ratio as a function of θ in the pre-exposed (in black), 2 h H*-exposed (in blue), and 4 h H*-exposed (in red) VC sample exposed to H* at 500 K. (a) V2p spectra, (b) C1s spectra of the VC sample, and (c) carbidic-C/V ratio. The carbidic fraction in the VC sample is stable, similar to the VC sample exposed to H* at 1000 K.

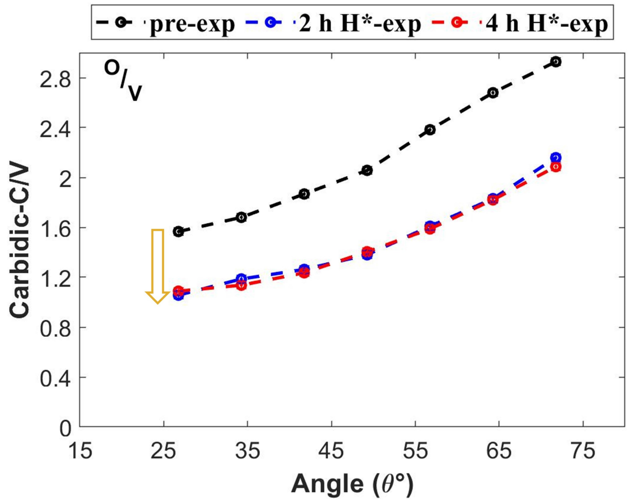

Figure S6. O/TM ratio as a function of θ in the pre-exposed (in black), 2 h H*-exposed (in blue), and 4 h H*-exposed (in red) VC sample exposed to H* at 500 K. Surface de-oxidation is less pronounced in the sample compared to the VC sample exposed to H* at 1000 K.

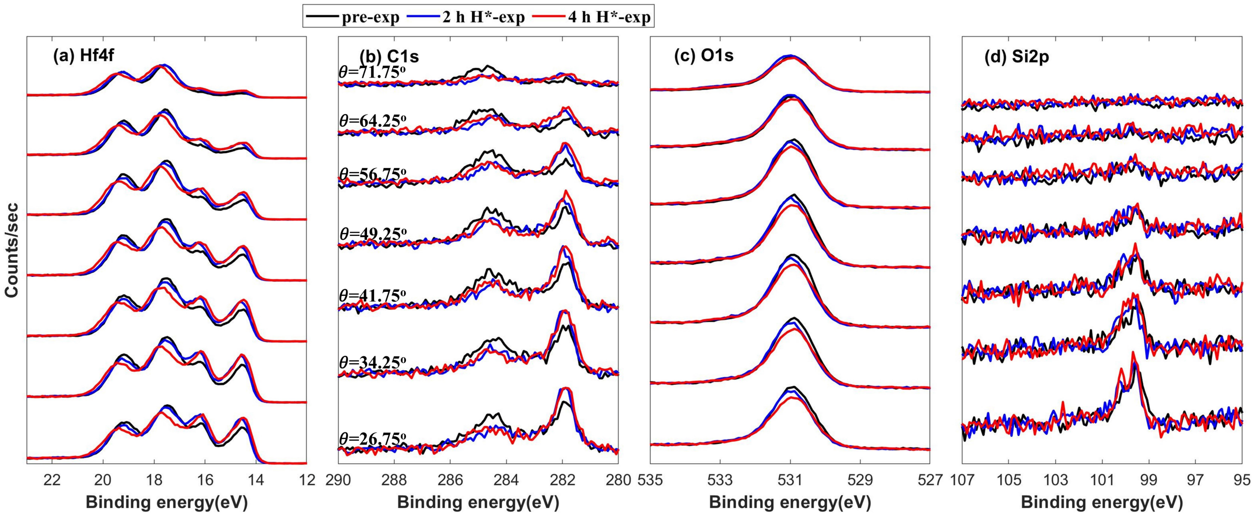

Figure S7. Comparison of the XPS spectra taken over the range of AR-XPS measurements of the pre-exposed (in black), 2 h H*-exposed (in blue), and 4 h H*-exposed (in red) HfC sample exposed to H* at 1000 K. (a) Hf4f, (b) C1s, (c) O1s, and (d) Si2p.

Table S1. Fitted peak positions in Hf4f and C1s XPS spectra of the per-exposed, 2 h H*-exposed, and 4 h H*-exposed HfC sample taken at $\theta = 34.25°$. Hf4f spectra are deconvoluted into Hf4f$_{7/2}$ and Hf4f$_{5/2}$ with an average separation of 1.66 eV.

Peak	Pre-exp	1st H*-exp	2nd H*-exp	Reference peaks
Hf4f$_{7/2}$ HfC	14.5	14.6	14.5	14.43/14.39[1] 14.1/13.9[2] 14.3[3]
Hf4f$_{7/2}$ HfOC/HfO$_{2-x}$	15.5	15.5	15.5	15.68 (Hf-O)[3]
Hf4f$_{7/2}$ HfO$_2$	17.5	17.6	17.8	17/16.9[2] 17.93/18.13[4]
C1s HfC	281.9	281.9	281.9	282.04/281.96[1] 282.6[3] 281.2[5]
C1s HfOC	283	283	283	282.5 (C-Hf*)[5]
C1s C-C	284.5	284.6	284.6	284.5[5] 284.4[6]
C1s C-O-C	286	-	-	286[6]
C1s O=C-C	288.5	-	-	288.5[6]

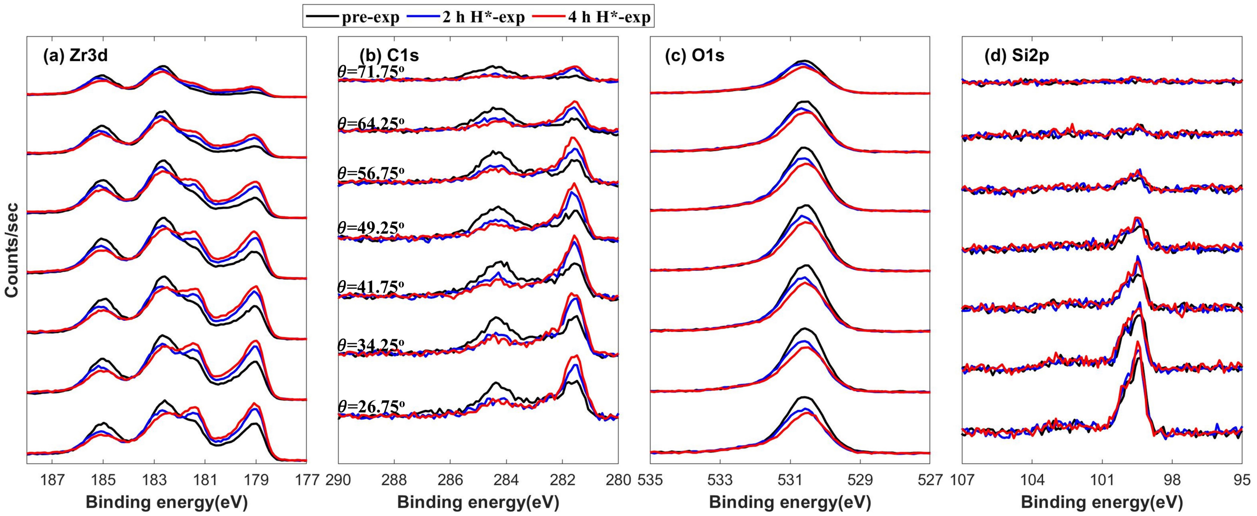

Figure S8. Comparison of the XPS spectra taken over the range of AR-XPS measurements of the pre-exposed (in black), 2 h H*-exposed (in blue), and 4 h H*-exposed (in red) ZrC sample exposed to H* at 1000 K. (a) Zr3d, (b) C1s, (c) O1s, and (d) Si2p.

Table S2. Fitted peak positions in Zr3d and C1s XPS spectra of the per-exposed, 2 h H*-exposed, and 4 h H*-exposed ZrC sample taken at $\theta = 34.25°$. Zr3d spectra are deconvoluted into $Zr3d_{5/2}$ and $Zr3d_{3/2}$ with an average separation of 2.4 eV.

Peak	Pre-exp	1st H*-exp	2nd H*-exp	Ref
$Zr3d_{5/2}$ ZrC	179	179	179	179.06[1] 178.7/179[2] 179.3[7]
$Zr3d_{5/2}$ ZrOC/ZrO_{2-x}	180.1	180.1	180.1	180.6/180.5/180.8 (suboxides)[7]
$Zr3d_{5/2}$ ZrO_2	182.6	182.6	182.6	184.5[1] 182.1/182.4/182.3[2] 182.2/182.4[7]
C1s ZrC	281.6	281.6	281.6	281.88/281.84[1] 281.7[8]
C1s ZrOC	282.5	282.5	282.5	282.6[8]
C1s C-C	284.4	284.4	284.4	284.3/285.2[8]
C1s C-O-C	286	-	-	286[6]
C1s O=C-C	288.5	-	-	288.5[6]

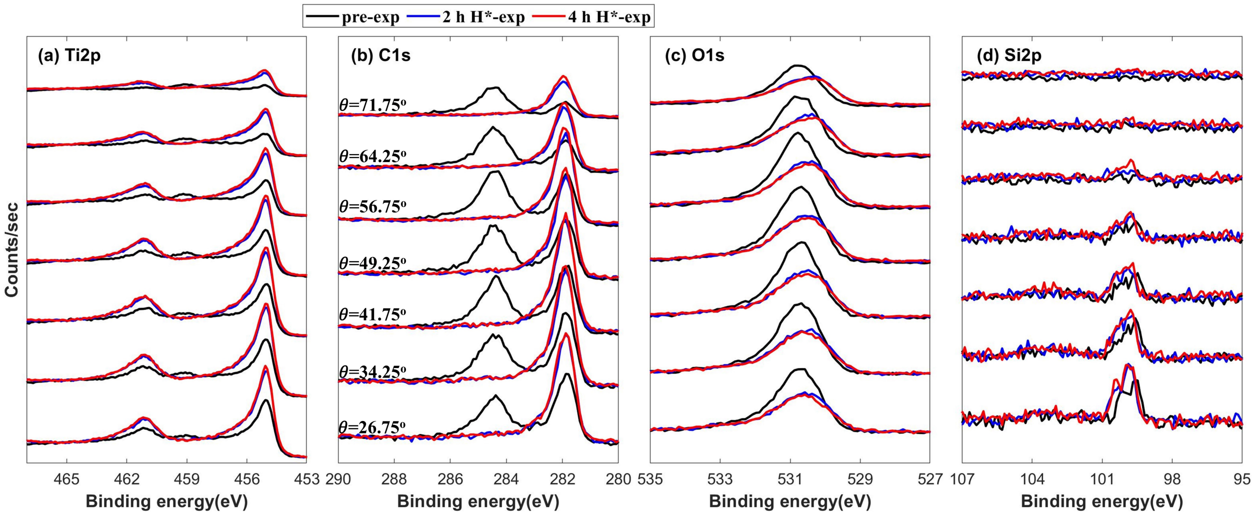

Figure S9. Comparison of the XPS spectra taken over the range of AR-XPS measurements of the pre-exposed (in black), 2 h H*-exposed (in blue), and 4 h time H*-exposed (in red) TiC sample exposed to H* at 1000 K. (a) Ti2p, (b) C1s, (c) O1s, and (d) Si2p.

Table S3. Fitted peak positions in Ti2p and C1s XPS spectra of the per-exposed, 2 h H*-exposed, and 4 h H*-exposed TiC sample taken at $\theta = 34.25°$. Ti2p spectra are deconvoluted into $Ti2p_{3/2}$ and $Ti2p_{1/2}$ with an average separation of 6 eV.

Peak	Pre-exp	1st H*-exp	2nd H*-exp	Ref
$Ti2p_{3/2}$ TiC	455.1	455.1	455.1	454.68/454.75[1] 455.2[2] 455.2[9] 455[10] 455.1[11]
$Ti2p_{3/2}$ TiOC/TiO_{2-x}	455.9	455.9	455.9	457.5/457.7[12]
$Ti2p_{3/2}$ TiO_2	458.9	458.9	458.9	459.2/459.4[12] 458.5[11]
C1s TiC	281.8	281.9	281.9	281.85/281.8[1] 281.5[13] 281.9[9] 281.5[14] 282[10]
C1s TiOC	282.5	282.5	282.5	282.9(C-Ti*)[9] 283.1[10]
C1s C-C	284.4	-	-	284.5/285.2[9] 284.1/284.9[14] 284.4[10]
C1s C-O-C	286	-	-	286[6]
C1s O=C-C	288.5	-	-	288.5[6]

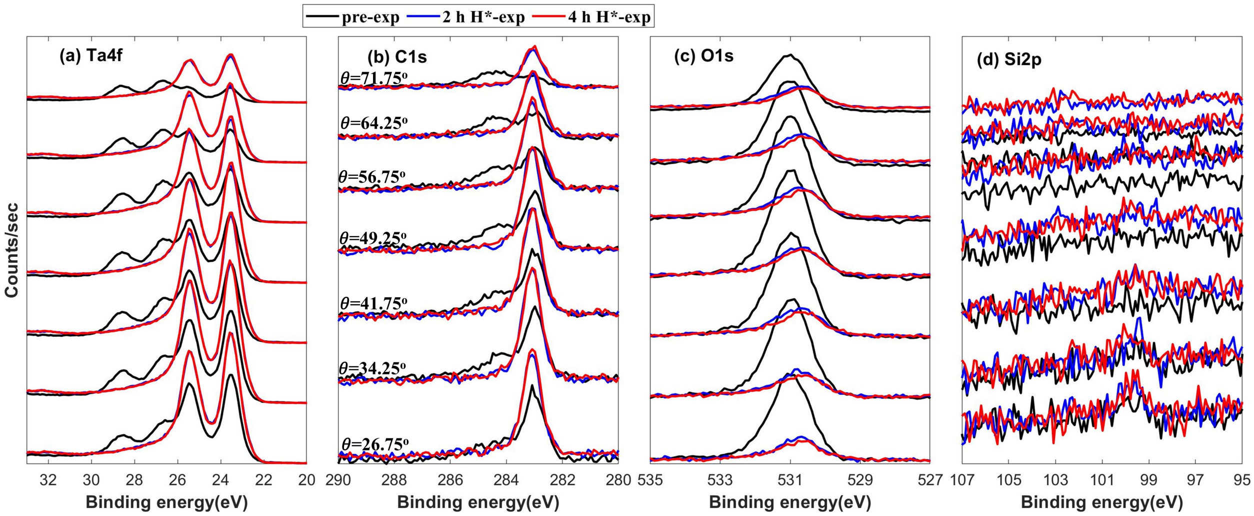

Figure S10. Comparison of the XPS spectra taken over the range of AR-XPS measurements of the pre-exposed (in black), 2 h H*-exposed (in blue), and 4 h time H*-exposed (in red) TaC sample exposed to H* at 1000 K. (a) Ta4f, (b) C1s, (c) O1s, and (d) Si2p.

Table S4. Fitted peak positions in Ta4f and C1s XPS spectra of the per-exposed, 2 h H*-exposed, and 4 h H*-exposed TaC sample taken at $\theta = 34.25°$. Ta4p spectra are deconvoluted into $Ta4p_{7/2}$ and $Ta4f_{5/2}$ with an average separation of 1.9 eV.

Peak	Pre-exp	1st H*-exp	2nd H*-exp	Ref
$Ta4f_{7/2}$ TaC	23.4	23.5	23.5	23.5[1] 23/23.3/23.2[2] 23.19[15]
$Ta4f_{7/2}$ TaOC/TaO_x	24.5	24.5	24.5	24.6/24[16]
$Ta4f_{7/2}$ Ta_2O_5	26.6	26.6	26.6	26.1[1] 26.3[17] 26.7/26.3[18]
O2s	23.6	23.6	23.6	23.6[19]
	Amplitude = 5% of O1s	Amplitude = 5% of O1s	Amplitude = 5% of O1s	Amplitude = 5% of O1s[20]
C1s TaC	283	283.1	283.1	283.11/283[1] 282.8[21] 282.8[15]
C1s TaOC				
C1s C-C	284.2	284.1	281.1	284.5/285.4[21]
C1s C-O-C	-	-	-	286[6]
C1s O=C-C	-	-	-	288.5[6]

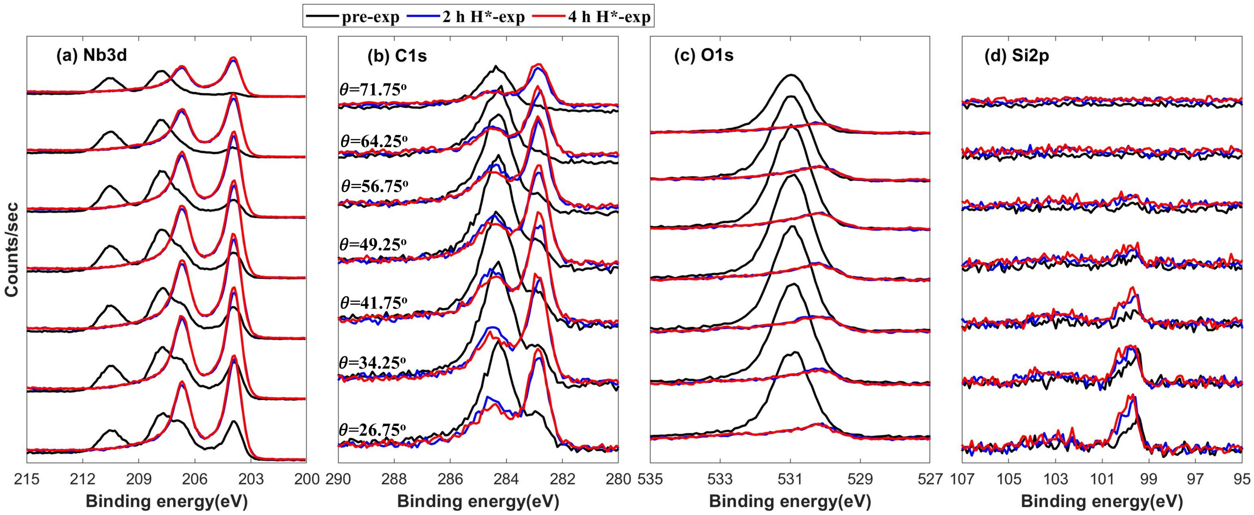

Figure S11. Comparison of the XPS spectra taken over the range of AR-XPS measurements of the pre-exposed (in black), 2 h H*-exposed (in blue), and 4 h H*-exposed (in red) NbC sample exposed to H* at 1000 K. (a) Nb3d, (b) C1s, (c) O1s, and (d) Si2p.

Table S5. Fitted peak positions in Nb3d and C1s XPS spectra of the per-exposed, 2 h H*-exposed, and 4 h H*-exposed NbC sample taken at $\theta = 34.25°$. Nb3d spectra are deconvoluted into $Nb3d_{5/2}$ and $Nb3d_{3/2}$ with an average separation of 2.7 eV.

Peak	Pre-exp	1st H*-exp	2nd H*-exp	Ref
$Nb3d_{5/2}$ NbC	203.9	203.9	203.9	203.91/203.75[1] 203.8/203.9[2]
$Nb3d_{5/2}$ NbOC/NbO_x	205.2	205.1	205.1	203.1 (NbO)[22] 203.5/203.7 (NbO)[23] 205.4 (NbO_2)[22]
$Nb3d_{5/2}$ Nb_2O_5	207.7	-	-	207.3/207.1[2] 207.7[24]
C1s NbC	282.9	282.8	282.9	282.82[1] 282.8[25] 282.3[22]
C1s NbOC				
C1s C-C	284.3	284.4	284.4	284.2/285.2[25]
C1s C-O-C	286	-	-	286[6]
C1s O=C-C	288.5	-	-	288.5[6]

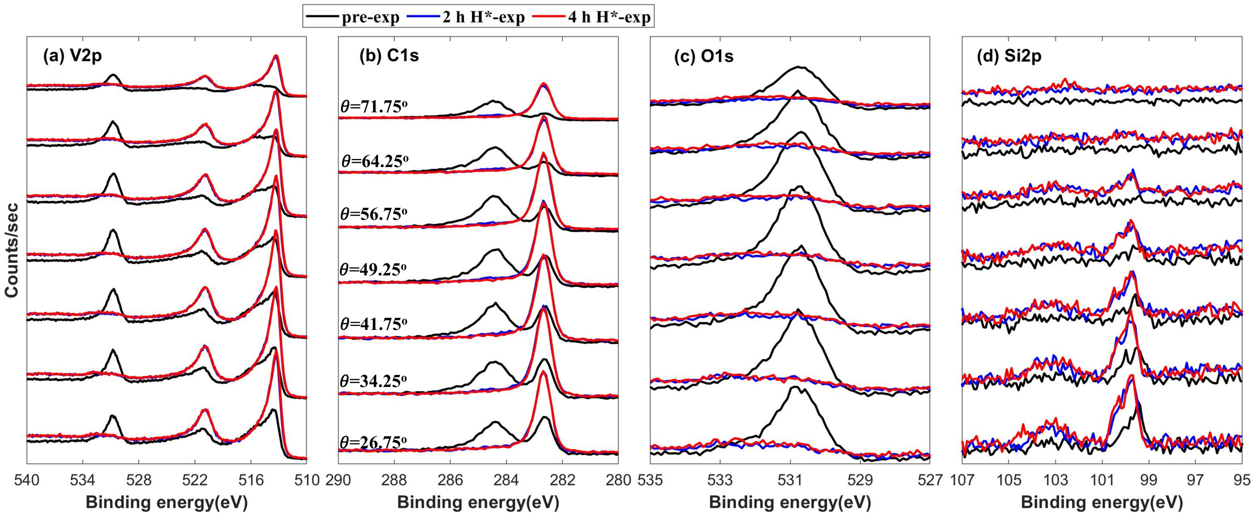

Figure S12. Comparison of the XPS spectra taken over the range of AR-XPS measurements of the pre-exposed (in black), 2 h H*-exposed (in blue), and 4 h H*-exposed (in red) VC sample exposed to H* at 1000 K. (a) V2p, (b) C1s, (c) O1s, and (d) Si2p.

Table S6. Fitted peak positions in V2p and C1s XPS spectra of the per-exposed, 2 h H*-exposed, and 4 h H*-exposed VC sample taken at $\theta = 34.25°$. V2p spectra are deconvoluted into $V2p_{3/2}$ and $V2p_{1/2}$ with an average separation of 7.5 eV.

Peak	Pre-exp	1st H*-exp	2nd H*-exp	Ref
$V2p_{3/2}$ VC	513.4	513.4	513.3	513.34/513.04[1] 513.9[26]
$V2p_{3/2}$ VOC/VO_x	515.3	514.7	514.7	515.3/515.85 (V_2O_3)[27]<br>515.65/516.4 (VO_2)[27]<br>516.2 (V2O3)/517.4 (VO_2)[26]
$V2p_{3/2}$ V_2O_5	517.6	517.6	517.6	516.9/517.5 (V_2O_5)[27]
C1s VC	282.7	282.7	282.7	282.67/282.51[1] 282.5[26]
C1s VOC				
C1s C-C	284.3	283.4	283.4	284.6[26]
C1s C-O-C	286	-	-	286[6] 286.3[26]
C1s O=C-C	288.5	-	-	288.5[6] 288.6[26]

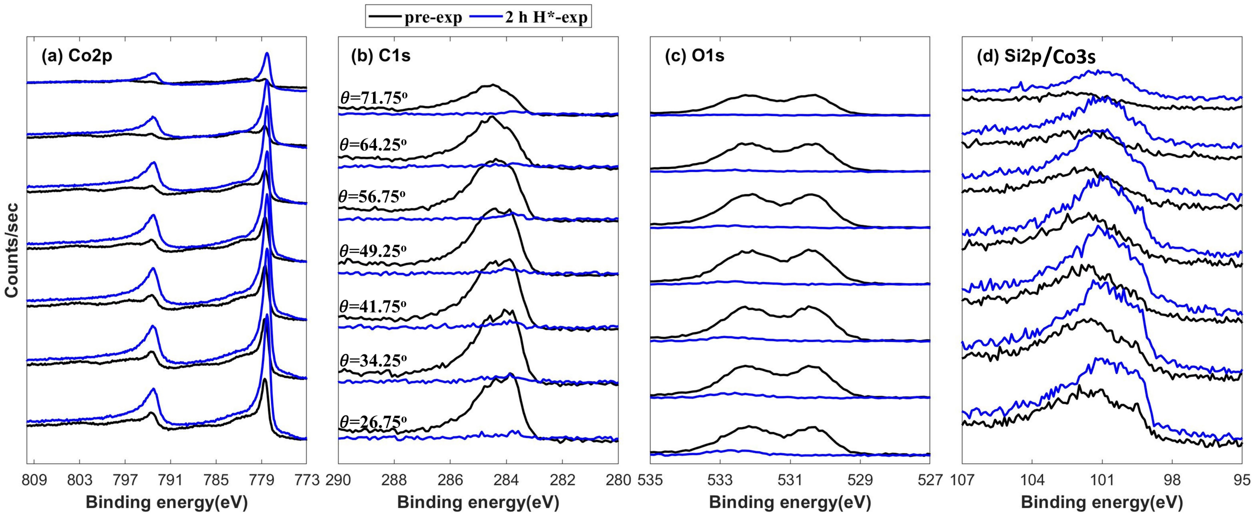

Figure S13. Comparison of the XPS spectra taken over the range of AR-XPS measurements of the pre-exposed (in black) and 2 h H*-exposed (in blue) H*-exposed (in red) Co_2C sample exposed to H* at 500 K. (a) Co2p, (b) C1s, (c) O1s, and (d) Si2p/Co3s.

Table S7. Peak positions in Co2p and C1s XPS spectra of the per-exposed and 2 h H*-exposed Co_2C sample taken at $\theta = 34.25°$.

Peak	Pre-exp	1st H*-exp	2nd H*-exp	Ref
$Co2p_{3/2}$ Co	778.3	778.3	-	778.85[28] 778.3[29]
$Co2p_{3/2}$ CoO	780	780	-	780.05[28]
$Co2p_{3/2}$ Co_2C	781	781	-	781.3[29]
C1s CoC	283.7	283.7	-	283.73[28]
C1s CoOC				
C1s C-C	284.6	284.6	-	284.4[28]
C1s C-O-C	286	286	-	
C1s O=C-C	288.6	288.6	-	288.2[28]

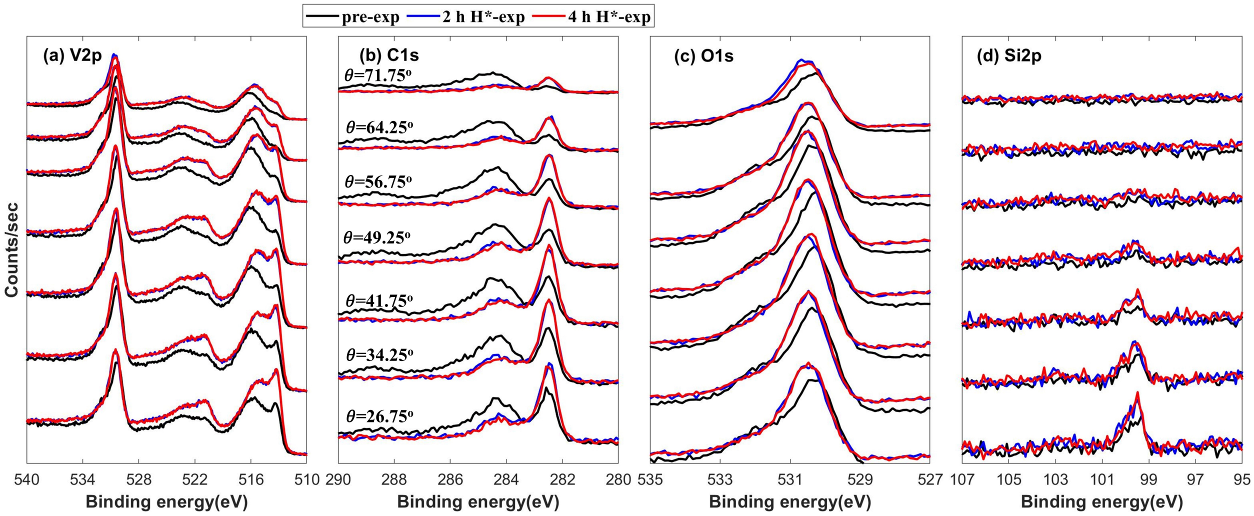

Figure S14. Comparison of the XPS spectra taken over the range of AR-XPS measurements of the pre-exposed (in black), 2 h H*-exposed (in blue), and 4 h H*-exposed (in red) VC sample exposed to H* at 500 K. (a) V2p, (b) C1s, (c) O1s, and (d) Si2p.

References

(1) Greczynski, G.; Primetzhofer, D.; Hultman, L. Reference binding energies of transition metal carbides by core-level x-ray photoelectron spectroscopy free from Ar+ etching artefacts. *Applied Surface Science* **2018**, *436*, 102–110.

(2) Fang, D.; Wang, C.; Lv, C.; Lv, Y.; Huang, G.; Yang, J.; Pan, S. XPS studies of surface oxidation of metal carbides. *Fullerenes, Nanotubes and Carbon Nanostructures* **2022**, *30*, 718–726.

(3) Pei, D.; Wang, L.; Ding, M.-H.; Hu, Z.-N.; Zhao, J.-Y.; Zhou, G.-Y.; Feng, Z.-R. The Effects of Substrate Bias on the Properties of HfC Coatings Deposited by RF Magnetron Sputtering. *Coatings* **2021**, *11*, 963.

(4) Morant, C.; Galan, L.; Sanz, J. An XPS study of the initial stages of oxidation of hafnium. *Surface and interface Analysis* **1990**, *16*, 304–308.

(5) Shuo, W.; Kan, Z.; Tao, A.; Chaoquan, H.; Qingnan, M.; Yuanzhi, M.; Mao, W.;

Weitao, Z. Structure, mechanical and tribological properties of HfCx films deposited by reactive magnetron sputtering. *Applied Surface Science* **2015**, *327*, 68–76.

(6) Calaway, M.; Fries, M. Adventitious Carbon on Primary Sample Containment Metal Surfaces. Lunar and Planetary Science Conference. 2015.

(7) Hauser, D.; Grießer, C.; Wernig, E.-M.; Götsch, T.; Bernardi, J.; Kunze-Liebhäuser, J.; Penner, S. The pervasive presence of oxygen in ZrC. *Surfaces and Interfaces* **2022**, *34*, 102373.

(8) Meng, Q. N.; Wen, M.; Mao, F.; Nedfors, N.; Jansson, U.; Zheng, W. Deposition and characterization of reactive magnetron sputtered zirconium carbide films. *Surface and Coatings Technology* **2013**, *232*, 876–883.

(9) Polychronopoulou, K.; Rebholz, C.; Baker, M.; Theodorou, L.; Demas, N.; Hinder, S.; Polycarpou, A.; Doumanidis, C.; Böbel, K. Nanostructure, mechanical and tribological properties of reactive magnetron sputtered TiCx coatings. *Diamond and related materials* **2008**, *17*, 2054–2061.

(10) Kuznetsov, M.; Borisov, S.; Shepatkovskii, O.; Veksler, Y. G.; Kozhevnikov, V. Investigation of TiC-C coatings by X-ray photoelectron spectroscopy. *Journal of surface investigation. x-ray, synchrotron and neutron techniques* **2009**, *3*, 331–337.

(11) Ignaszak, A.; Song, C.; Zhu, W.; Zhang, J.; Bauer, A.; Baker, R.; Neburchilov, V.; Ye, S.; Campbell, S. Titanium carbide and its core-shelled derivative TiC@ TiO2 as catalyst supports for proton exchange membrane fuel cells. *Electrochimica acta* **2012**, *69*, 397–405.

(12) Daughtry, J.; Alotabi, A. S.; Howard-Fabretto, L.; Andersson, G. G. Composition and properties of RF-sputter deposited titanium dioxide thin films. *Nanoscale Advances* **2021**, *3*, 1077–1086.

(13) Gulbiński, W.; Mathur, S.; Shen, H.; Suszko, T.; Gilewicz, A.; Warcholiński, B. Evaluation of phase, composition, microstructure and properties in TiC/aC: H thin films deposited by magnetron sputtering. *Applied Surface Science* **2005**, *239*, 302–310.

(14) Aguzzoli, C.; Figueroa, C.; Soares, G.; Baumvol, I. Physicochemical and structural characteristics of TiC and VC thin films deposited by DC reactive magnetron sputtering. *Journal of materials science* **2010**, *45*, 4994–5001.

(15) Vargas, M.; Castillo, H.; Restrepo-Parra, E.; De La Cruz, W. Stoichiometry behavior of TaN, TaCN and TaC thin films produced by magnetron sputtering. *Applied surface science* **2013**, *279*, 7–12.

(16) Kasatikov, S.; Filatova, E.; Sakhonenkov, S.; Konashuk, A.; Makarova, A. Relationship between Ta oxidation state and its local atomic coordination symmetry in a wide range of oxygen nonstoichiometry extent of TaO x. *The Journal of Physical Chemistry C* **2019**, *123*, 6849–6860.

(17) Aihaiti, L.; Tuokedaerhan, K.; Sadeh, B.; Zhang, M.; Qian, S. X.; Mijiti, A. Electrical and microstructural properties of Ta-C thin films for metal gate. *Materials Research Express* **2020**, *7*, 076402.

(18) Moo, J. G. S.; Awaludin, Z.; Okajima, T.; Ohsaka, T. An XPS depth-profile study on electrochemically deposited TaO x. *Journal of Solid State Electrochemistry* **2013**, *17*, 3115–3123.

(19) Lamour, P.; Fioux, P.; Ponche, A.; Nardin, M.; Vallat, M.-F.; Dugay, P.; Brun, J.-P.; Moreaud, N.; Pinvidic, J.-M. Direct measurement of the nitrogen content by XPS in self-passivated TaNx thin films. *Surface and Interface Analysis: An International Journal devoted to the development and application of techniques for the analysis of surfaces, interfaces and thin films* **2008**, *40*, 1430–1437.

(20) McLellan, R. A.; Dutta, A.; Zhou, C.; Jia, Y.; Weiland, C.; Gui, X.; Place, A. P.; Crowley, K. D.; Le, X. H.; Madhavan, T. et al. Chemical profiles of the oxides on tantalum in state of the art superconducting circuits. *Advanced Science* **2023**, *10*, 2300921.

(21) Luo, H.; Yazdi, M. A. P.; Chen, S.-C.; Sun, H.; Gao, F.; Heintz, O.; de Monteynard, A.; Sanchette, F.; Billard, A. Structure, mechanical and tribological properties, and oxidation resistance of TaC/aC: H films deposited by high power impulse magnetron sputtering. *Ceramics International* **2020**, *46*, 24986–25000.

(22) Kowalski, K.; Bernasik, A.; Singer, W.; Singer, X.; Camra, J. In situXPS investigation of the baking effect on the surface oxide structure formed on niobium sheets used for superconducting RF cavity production. Proc. of the 11th Workshop on RF Superconductivity, Travemünde, Germany. 2003.

(23) Hu, Z.; Li, Y.; Ji, M.; Wu, J. The interaction of oxygen with niobium studied by XPS and UPS. *Solid state communications* **1989**, *71*, 849–852.

(24) Chen, L.; Sun, Q.-Q.; Gu, J.-J.; Xu, Y.; Ding, S.-J.; Zhang, D. W. Bipolar resistive switching characteristics of atomic layer deposited Nb2O5 thin films for nonvolatile memory application. *Current Applied Physics* **2011**, *11*, 849–852.

(25) Zhang, K.; Wen, M.; Cheng, G.; Li, X.; Meng, Q.; Lian, J.; Zheng, W. Reactive magnetron sputtering deposition and characterization of niobium carbide films. *Vacuum* **2014**, *99*, 233–241.

(26) Wu, H.; Cao, Z.; Zuo, C.; Zhang, X. A simple and novel route for synthesis of vanadium carbide nanoparticles. *Res. Rev.: J. Mater. Sci.* **2019**, *6*, 251–258.

(27) Hryha, E.; Rutqvist, E.; Nyborg, L. Stoichiometric vanadium oxides studied by XPS. *Surface and interface analysis* **2012**, *44*, 1022–1025.

(28) Ma, X.; Li, K.; Zhang, X.; Wei, B.; Yang, H.; Liu, L.; Zhang, M.; Zhang, X.; Chen, Y. The surface engineering of cobalt carbide spheres through N, B co-doping achieved by room-temperature in situ anchoring effects for active and durable multifunctional electrocatalysts. *Journal of Materials Chemistry A* **2019**, *7*, 14904–14915.

(29) Yang, F.; Zhao, H.; Wang, W.; Liu, Q.; Liu, X.; Hu, Y.; Zhang, X.; Zhu, S.; He, D.; Xu, Y. et al. Carbon-involved near-surface evolution of cobalt nanocatalysts: an in situ study. *CCS Chemistry* **2021**, *3*, 154–167.
